# Purification, characterization and anti-aging capacity of mycelia zinc polysaccharide by *Lentinus edodes* SD-08

**DOI:** 10.1186/s12906-015-0630-7

**Published:** 2015-04-09

**Authors:** Liqin Wang, Cuiqin Wang, Xia Gao, Nuo Xu, Lin Lin, Huajie Zhao, Shouhua Jia, Le Jia

**Affiliations:** College of Life Science, Shandong Agricultural University, Daizong Street 61, Taian, Shandong 271018 PR China; Government Hospital of Yantai, Yuxi Road 16, Yantai, Shandong 264000 PR China; Shandong Agricultural Technology Extending Station, Jinan, Shandong 250100 PR China; College of Chemistry and Material Science, Shandong Agricultural University, Daizong Street 61, Taian, Shandong 271018 PR China

**Keywords:** Anti-aging, Chemical bonds, *Lentinus edodes* SD-08, Mycelia zinc polysaccharides, Monosaccharide composition

## Abstract

**Background:**

In the modern society, aging had been a major problem. People may rely on many medicines to delay it. However, lots of medicines were chemosynthetic, and they would do a bad side-effect on human body. Microbial sources could be used as a potential means of producing natural antioxidants. *Lentinus edodes,* commercial obtained in daily life, had recently become more attractive in physiological research. Zinc was now considered as a major element in assuring the correct functioning of an organism and essential for maintaining coordination of the major homeostatic networks. To investigate the bioconversion of zinc and the physiological effects of their complex (MZPS), the present studies were processed.

**Methods:**

Mycelia polysaccharides (MPS) and mycelia zinc polysaccharides (MZPS) of *Lentinus edodes* SD-08 were extracted by hot water leaching and purified by DEAE-52 cellulose anion-exchange column chromatography separately. The zinc content was determined by flame atomic absorption spectrometry. The evaluation of monosaccharide compositions and proportions used gas chromatogram. The analysis of molecular weight used HPGPC chromatogram. The typical structure of polysaccharide was evaluated by IR spectrum. The antioxidant activities *in vitro* measured through reducing power, the scavenging effects on hydroxyl radical and 1,1-diphenyl-2-picrylhydrazyl (DPPH) radicals. The anti-aging activities *in vivo* measured through the total antioxidant capacity (T-AOC), GSH peroxide (GSH-Px), superoxide dismutase (SOD) and the contents of malondialdehyde (MDA).

**Results:**

MPS and MZPS of *Lentinus edodes* SD-08 were extracted and purified by DEAE-52 cellulose anion-exchange column chromatography separately, and four fractions (MPS-1, MPS-2, MZPS-1 and MZPS-2) were obtained. In addition, MPS composing of rhamnose, arabinose and mannose (molar proportion = 1.75:1.00:3.02) and MZPS containing rhamnose, arabinose, mannose and glucose (molar proportion = 7.19:2.26:1.00:8.39) were investigated by gas chromatography. Infrared spectrum analysis indicated that there were C-H, C=O and -CH_2_ bonds in MPS and MZPS. MPS also had the typical absorption of -NH_3_^+^, -NH_2_ and -COOH. Compared with MPS, MZPS showed *in vitro* positive rising of reducing power and certain scavenging effects on hydroxyl radical and 1,1-diphenyl-2-picrylhydrazyl (DPPH) radicals. MZPS were found to upregulate *in vivo* the anti-aging activities of total antioxidant capacity (T-AOC), GSH peroxide (GSH-Px), superoxide dismutase (SOD), and decrease the contents of malondialdehyde (MDA).

**Conclusions:**

MZPS effectively showed potential anti-aging activities *in vivo* and antioxidant activities *in vitro*, and the molecular constituents, chemical bonds and functional groups of MZPS were superior to MPS, suggesting that the MZPS of *L. edodes* SD-08 could be used as a potential natural antioxidant.

## Background

Mushrooms play an important role in diet and have recently become attractive as sources for the development of drugs, so many researchers paid much attention on them [1,2]. In the global market, *Lentinus edodes*, as its nutritional value and medical application, has been the second most popular edible mushroom [3,4]. Lentinan, a β-glucan, is the most important polysaccharide isolated from *L. edodes*, because of its antioxidant activity, immunomodulatory and antitumor effects [5-7]. Although lentinan has been known for its kinds of bioactivities, there were obstacles for its development as clinic, such as its extraction, purification, chain conformation and the relationship between structure and biological activities. Therefore, a basic understanding of the chemical structure, the chain conformation and bioactivities of lentinan is essential for its successful applications in disease prevention and treatment.

Zinc (Zn) is one of the most important trace elements in human body and now considered as a major element in assuring the correct functioning of an organism. Zinc is essential for maintaining coordination of the major homeostatic networks, i.e., the nervous, neuroendocrine and immune systems, adds support to a new theoretical approach to explain aging processes [8] during the organism’s entire life [9]. Zinc is important for the activity of at least 90 enzymes which participate in all the major metabolic pathways. Over 40 metalloenzymes exist in which Zn is bound tightly to the apoenzyme in specific stoichiometric ratios and which Zn serves one or more structural, regulatory or catalytic functions [10]. In addition to its functions in enzymes, zinc participates in the metabolism of nucleic acids and synthesis of proteins [11]. In normal diet, some substances, especially phytic acid, have an inhibitory effect on the utilization (bioavailability) of Zn in the human digestive tract. Combined with the influence of microorganisms, the absorption rate of zinc is lower than that in meat, because of phytic acid zinc which is generated by phytic acid and zinc is not easily dissolved, leading to the reducing of the absorption of zinc. So zinc deficiency has become a world nutritional problem since over 25% of the world’s population is at risk of zinc deficiency [12]. Inorganic zinc is defected by its difficulty in absorption and unsuitability for human and animal. Organic zinc polysaccharide transformed by edible medicinal mushrooms hyphae cell’s metabolism, is more conducive to the immune function of organic zinc and resistant function of edible-medicinal mushrooms.

In this paper, *Lentinus edodes* SD-08 was cultured in liquid media using ZnSO_4_ as zinc supplement_._ The mycelia polysaccharide (MPS) and mycelia zinc polysaccharide (MZPS) of *L. edodes* SD-08 were separately purified, and four fractions (MPS-1, MPS-2, MZPS-1 and MZPS-2) were obtained by DEAE-52 cellulose anion-exchange column chromatography. Molecular constituents, chemical bonds and functional groups of MPS or MZPS were investigated. The *in vivo* anti-aging capacities and *in vitro* antioxidant activities of MZPS were evaluated.

## Methods

### Microorganism and cultural conditions

*L. edodes* SD-08 was from our laboratory at 4°C and maintained on synthetic potato dextrose agar (PDA) slants. The liquid fermentation technology was used to produce *L. edodes* SD-08 mycelia. Each 1 L erlenmeyer flask, containing 500 mL of liquid medium (glucose 20 g/L, peptone 3 g/L, yeast extract 4 g/L, KH_2_PO_4_ 1 g/L, MgSO_4_ 1 g/L and ZnSO_4_ · 7H_2_O 0.15 g/L), was inoculated with a 1 cm^2^ mycelial block of *L. edodes* SD-08 from the solid media. The liquid culture was grown at 25°C with a shaking of 160 r/min for 14 d.

### Chemicals

1,1-dipheny-l,2-picrylhydrazyl (DPPH) was from Sigma Chemicals Company (St. Louis, USA). DEAE-52 cellulose anion-exchange was from Whatman Chemicals Company (UK). All other chemicals used in this experiment were analytical reagent grade and purchased from local chemical suppliers in China.

### Extraction of polysaccharides

Three times of distilled water were added to a beaker with mycelia powder 100 g, and the mixture was sonicated for 600 s using sonifier cell disrupter (Scientz-IID, Ningbo Scientz Biotechnology. Co., Ltd. CHN). The homogenate was heated at 85°C for 3 h and centrifuged at 3000 r/min for 10 min. The supernatant was collected and the above process was repeated three times. Four times of alcohol (95%, v/v) was mixed with the supernatant and kept at –4°C for 12 h. After centrifuged at 3000 r/min for 20 min, the precipitation (MPS or MZPS) was lyophilized. The content of MPS or MZPS was determined by phenol–sulfuric acid method, using glucose as standard [13].

### Separation of MPS and MZPS

MPS and MZPS were separately fractionated by DEAE-52 cellulose anion-exchange column (2.5 × 65 cm). When polysaccharides were poured into the DEAE-52 cellulose, DEAE-52 could combine the polysaccharides which could be eluted by the increasing intensity of NaCl (0.1 M, 0.3 M, 0.5 M, and 1 M NaCl), and only strong enough ionic force could elute the different fragments [14]. The major polysaccharide fractions were collected with a fraction collector. Sugar contents of each tube were assayed by the phenol–sulfuric acid method using glucose as standard [13].

### Antioxidant activities *in vitro*

#### Reducing power assay

The reducing power of polysaccharides was measured estimated according to the method of Oyaizu [15] with a slight modification. The reaction mixtures contained 2.5 mL phosphate buffer (pH 6.6, 0.2 M), 1 mL potassium ferricyanide (1%, w/v) and the polysaccharide (0.5-5 g/L). After incubating at 50°C for 20 min, 2 mL of trichloroacetic acid (10%, w/v) and 1.2 mL of FeCl_3_ (0.1%, w/v) were added to the mixture for terminating the reaction, and then centrifuged at 1200 r/min for 10 min. After incubating at 25°C for 15 min, the absorbances of the samples were measured at 700 nm.

#### Hydroxyl radical scavenging assay

Hydroxyl radical scavenging activity was measured according to the method of Winterbourn and Sutton [16]. One milliliter sample solution was mixed with 1 mL of 8.8 mM H_2_O_2_, 1.0 mL of 9 mM FeSO_4_, and 1 mL of 9 mM salicylic ethanol solution. After incubating at 37°C for 30 min, the absorbances of polysaccharides were then measured at 510 nm using UV-4802 double-beam UV-spectrophotometer. The hydroxyl radical scavenging activity was expressed as:

1 $$ \mathrm{Scavenging}\ \mathrm{rate}\ \left(\%\right) = \left[\left({\mathrm{A}}_0\hbox{--} {\mathrm{A}}_1\right)/{\mathrm{A}}_0\right] \times 100 $$

Where A_0_ is the absorbance of the blank and A_1_ is the absorbance of the sample.

#### DPPH scavenging assay

The experimental of DPPH scavenging activity of MPS and MZPS were according to the method of Liu and Zhao [17]. The reaction mixture contained 2 mL of anhydrous ethyl alcohol, 0.2 mM DPPH and 2 mL of the polysaccharides (0.1–1 g/L). The solution was incubated at 25°C for 30 min in the dark, and the absorbance of polysaccharides was determined at 517 nm. Absolute ethanol was used as the blank. The antioxidant activity of polysaccharides was evaluated according to the following formula:

2 $$ \mathrm{Scavenging}\ \mathrm{rate}\;\left(\%\right) = \left[1\hbox{--} \left({\mathrm{A}}_{\mathrm{i}}\hbox{--} {\mathrm{A}}_{\mathrm{j}}\right)/{\mathrm{A}}_{\mathrm{c}}\right] \times 100\% $$

Where A_c_ is the absorbance of 2 mL DPPH and 2 mL absolute ethanol, A_i_ and A_j_ is the absorbance of 2 mL sample and 2 mL DPPH or absolute ethanol.

### Analysis of polysaccharides

#### Determination of zinc content

MPS and MZPS (0.1 g) were weighed severally, and then added 5 mL HNO_3_ and 2 mL HClO_4_ and let the mixture standing overnight. The mixture was boiled until the cooking liquor clear. After digestion of the samples, the mixture was diluted to 25 mL. The zinc content was measured by using flame atomic absorptions spectrometric method (novAA300, Analytik-Jena, Germany).

#### Analysis of monosaccharide compositions

Monosaccharide composition was determined by gas chromatography (GC) (GC-2010, Shimadzu, Japan) equipped with a capillary column of Rtx-1 (30 mm × 0.25 mm × 0.25 μm) using the method in the literature [18] with slight modifications. Sugar identification was done by comparison with standard monosaccharides (rhamnose, ribose, arabinose, xylose, inositol , mannose, glucose and galactose) (Sigma, USA).

#### Determination of molecular weight

The molecular weight and homogeneity of MZPS were determined by high performance liquid gel permeation chromatography (HPGPC) that was operated with a HPLC system (1260, Agilent Technologies, USA) equipped with a SHODEX SB-806HQ column (8.0 mm × 300 mm) and a refractive index detector. The injection volume was 100 μL. NaCl aqueous solution (0.2M) was used as mobile phase at a flow rate of 0.5 mL/min, and the column temperature was maintained at 35°C. A series of standard dextrans with known Mw (molecular weights) values were used to make the calibration curve, and the regression equation of logMw against elution time (ET) was as follows: logMw = –0.3429ET + 11.975, R2 = 0.9991. Molecular weights was analysed by Agilent GPC software.

#### Infrared spectral analysis

The IR spectrum of the polysaccharides was carried out using a Fourier transform infrared spectrophotometer (FTIR, Bruker, Germany) equipped with OPUS 3.1 software. The components of MPS or MZPS were ground with KBr powder and then pressed into pellets for FTIR measurement at the frequency range of 4000–500 cm^-1^ [19].

### Animal experiments

Forty-two male mice (Kunming strain), weighing 20 ± 2 g were provided by Center for Animal Testing of Shandong Lukang Drugs Group Ltd. (Jining, China) and housed in stainless steel cages under controlled conditions (temperature 22 ± 1°C, humidity 60 to 65%, lights on 12 h every day) with free access to standard food. After a 3 day acclimatization period, all animals were randomly divided into six groups (7 in each group). Model group (MG) was intraperitoneal injected with 0.2 mL D-galactose (25%, w/v). Blank group (BG) was gavaged with 0.2 mL distilled water. Low MPS (LMPS) and high MPS (HMPS) were perfused 200 and 800 mg/kg body weight of MPS, respectively. Low MZPS (LMZPS) and high MZPS (HMZPS) were perfused 200 and 800 mg/kg body weight of MZPS, respectively. LMPS, HMPS, LMZPS and HMZPS were intraperitoneal injected with 0.2 mL D-galactose (25%, w/v) simultaneously. The mice were allowed to have free access to water and food for 30 d. The experiments were performed under the guidance of Committee of Shandong Agricultural University.

At the end of the experiment, all animals were sacrificed under ether anesthesia, and blood samples were taken from the retrobulbar vein with a vacutainer and anticoagulated by heparin (stored at –80°C). The hearts, livers, spleens, and kidneys were rapidly removed, weighed, and homogenized (1:9, w/v) immediately in 0.2 M phosphate buffer (4°C, pH 7.4), respectively. The homogenates were centrifuged (6000 r/min) at 4°C for 20 min and the supernatants were stored at –20°C for the evaluation of the activities of the enzyme and nonenzyme total antioxidant capacity (T-AOC), superoxide dismutase (SOD), glutathione peroxidase (GSH-Px) and the content of malonaldehyde (MDA).

#### Anti-aging activities *in vivo*

The T-AOC and SOD of serum and liver were analyzed using commercial kits (Nanjing Jiancheng Bioengineering Institute, Nanjing, China).

The activity of GSH-Px was measured as described by Flohé and Günzler [20]. The reaction mixture consisted of 50 mM potassium phosphate buffer [pH 7.0], 0.5 mM EDTA, 1 mM NaN_3_, 0.15 mM NADPH, 1 mM GSH, and 2.4 U/mL of glutathione reductase (GR). The reaction was initiated by adding 0.15 mM H_2_O_2_. The rate of nicotinamide adenine dinucleotide phosphate (NADPH) consumption was recorded at 340 nm. The activity of GSH-Px was expressed as mM of NADPH oxidized per minute per milligram of tissue or micromoles per minute per milliliter of blood.

The content of MDA was measured according to the method of Zhao et al. [21] with a slight modification. The mixture contained 0.2 mL tissue sample and 2 mL of 0.6% thiobarbituric acid (TBA, w/v), after heating in boiling water for 15 min, cooling rapidly, then centrifuged at 3000 r/min for 10 min, and the supernatant was used for the determination of MDA level.

3 $$ \mathrm{Content}\ \mathrm{of}\ \mathrm{M}\mathrm{D}\mathrm{A}\ \left(\mu \mathrm{mol}/\mathrm{L}\right)=6.45 \times \left({\mathrm{A}}_0\hbox{--} {\mathrm{A}}_1\right)\hbox{--} 0.56\times {\mathrm{A}}_2 $$

Where A_0_ is the absorbance at 532 nm, A_1_ is the absorbance at 600 nm, and A_2_ is the absorbance at 450 nm.

### Statistical analysis

All experiments were carried out in triplicates and results were recorded as means ± standard deviation (S.D.). P < 0.05 was considered to be statistically significant. Determination of parameters of MPS and MZPS were processed and analyzed using SPSS 16.0.

## Results

### Purification and antioxidant ability in vitro

**Figure 1:**
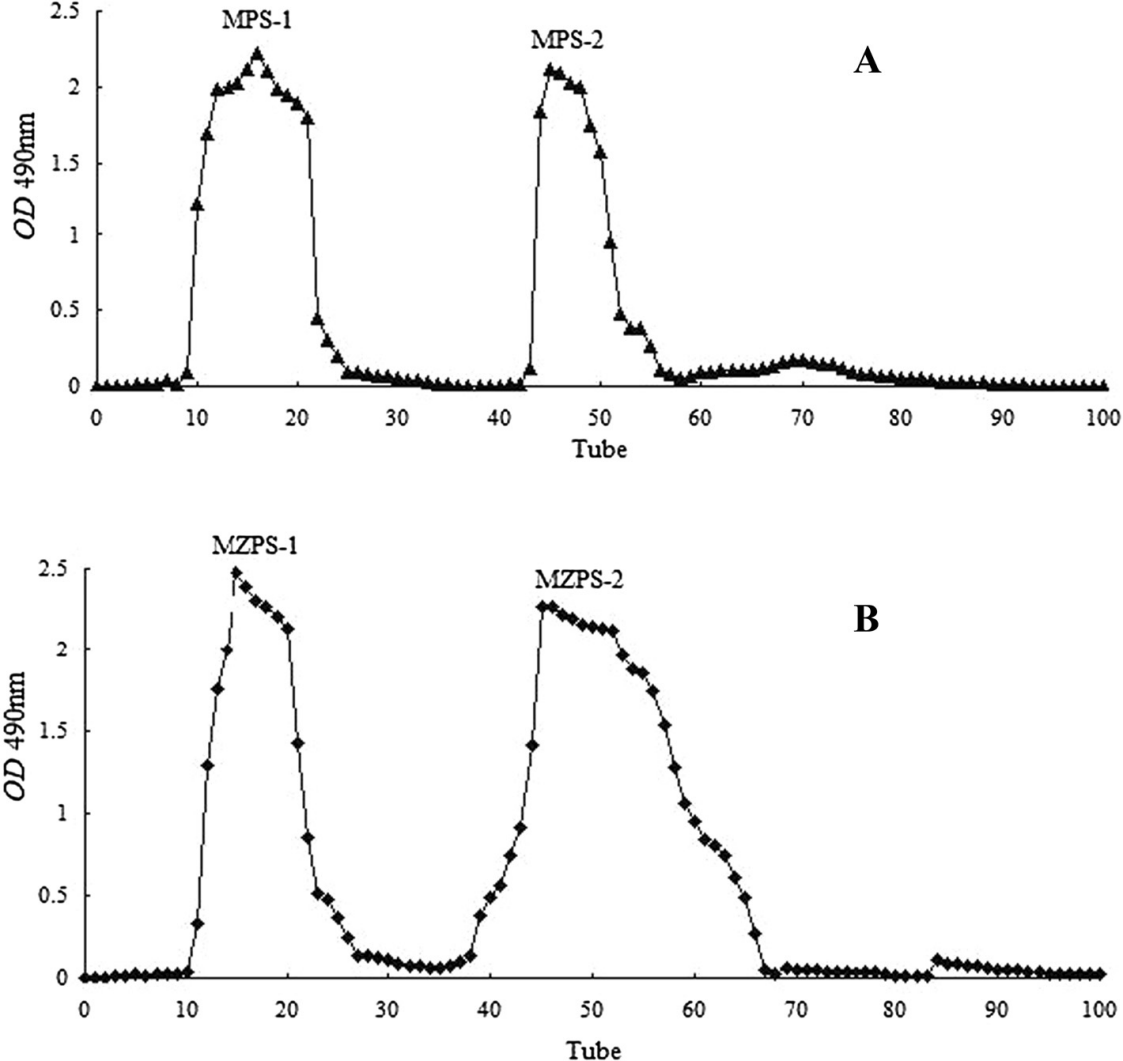
**DEAE–52 cellulose anion–exchange column chromatogram. A** MPS and **B** MZPS.

The results of MPS and MZPS purification were shown in Figure 1, and four fractions were obtained by DEAE-52 cellulose chromatography, namely MPS-1, MPS-2, MZPS-1 and MZPS-2.

As shown in Table 1, the reducing power (absorbance at 700 nm) of MPS and MZPS exhibited a dose-dependent effect. The reducing power of MZPS at 1000 mg/L was 0.28 (P < 0.01), which was 23.68 ± 0.01% higher than that of MPS (0.23 ± 0.01, P < 0.01). The reducing power of MZPS-1 at 300 mg/L was 0.25 ± 0.01 (P < 0.01), which was not only 6.10 ± 0.01% higher than that of MZPS-2 (0.19 ± 0.01, P < 0.01), but also higher than that of MPS-1 (0.03 ± 0.01, P < 0.01) and MPS-2 (0.05 ± 0.01, P < 0.01). The reducing power value of MZPS-1 was also much higher than 0.1 of *P. baumii* [22] and 0.38 of *A. bisporus* [23], indicating that the MZPS-1 of *L. edodes* SD-08 had the higher antioxidant capacities *in vitro*.

**Table 1:** **Reducing power of MPS and MZPS**

**Concentration [mg/L]**	**MPS**	**MPS–1**	**MPS–2**	**MZPS**	**MZPS–1**	**MZPS–2**
0	0	0	0	0	0	0
50	—	0.016	0.024	—	0.071	0.077
100	0.065	0.019	0.031	0.220	0.094	0.112
150	—	0.025	0.043	—	0.164	0.131
200	0.095	0.024	0.041	0.226	0.190	0.137
250	—	0.026	0.042	—	0.223	0.143
300	0.134	0.034	0.05	0.229	0.253	0.192
400	0.162	—	—	0.229	—	—
500	0.18	—	—	0.242	—	—
600	0.183	—	—	0.244	—	—
700	0.187	—	—	0.245	—	—
800	0.196	—	—	0.257	—	—
900	0.205	—	—	0.259	—	—
1000	0.228	—	—	0.282	—	—

“—”: Not determination.

As shown in Table 2, the scavenging rate reached 66.20 ± 0.01% (P < 0.01) at a dosage of 1000 mg/L, which was 7.22 ± 0.02% higher than that of MPS (58.99 ± 0.01%, P < 0.05). The scavenging rate of MZPS-1 (98.90 ± 0.01%, P < 0.05) and MZPS-2 (88.70 ± 0.01%, P < 0.05) were also much higher than that of MZPS at the concentration of 300 mg/L. The scavenging rate of MZPS-1 and MZPS-2 was higher than 44.10% of *P. nebrodensis* [24], 28.30% of *C. militaris* [25]*,* 38.10% of *T. matsutake* [26], and 0.72% of *P. ostreatus* [27], respectively. The results showed that the scavenging rate of MZPS of *L. edodes* SD-08, especially MZPS-1, significantly affects the scavenging of hydroxyl radical.

**Table 2:** **Hydroxyl radical scavenging rate of MPS and MZPS**

**Concentration [mg/L]**	**MPS**	**MPS–1**	**MPS–2**	**MZPS**	**MZPS–1**	**MZPS–2**
0	0	0	0	0	0	0
50	—	0.005	0.037	—	0.301	0.296
100	0.154	0.014	0.106	0.158	0.454	0.405
150	—	0.045	0.134	—	0.536	0.437
200	0.226	0.053	0.200	0.241	0.859	0.582
250	—	0.055	0.263	—	0.894	0.814
300	0.245	0.090	0.333	0.271	0.989	0.887
400	0.308	—	—	0.359	—	—
500	0.334	—	—	0.403	—	—
600	0.385	—	—	0.437	—	—
700	0.431	—	—	0.503	—	—
800	0.509	—	—	0.578	—	—
900	0.524	—	—	0.593	—	—
1000	0.590	—	—	0.662	—	—

“—”: Not determination.

It can be seen from Table 3 that the DPPH scavenging ability of MZPS at 1000 mg/L was 77.33 ± 0.01% (P < 0.01), 63.56 ± 0.03% higher than that of MPS (13.77 ± 0.01%, P < 0.01). MZPS-2 had the highest scavenging rate (75.50 ± 0.01%, P < 0.01) at 300 mg/L, which was higher than 30.80% of *C. militaris* [28] and 17.80% of *C. sinensis* [29]. The DPPH scavenging results revealed that the MZPS probably contained substances that were proton donors and could react with free radicals to convert them to stable diamagnetic molecules.

**Table 3:** **DPPH scavenging rate of MPS and MZPS**

**Concentration [mg/L]**	**MPS**	**MPS–1**	**MPS–2**	**MZPS**	**MZPS–1**	**MZPS–2**
0	0	0	0	0	0	0
50	—	0.036	0.046	—	0.020	0.031
100	0.026	0.042	0.048	0.120	0.050	0.099
150	—	0.069	0.076	—	0.083	0.152
200	0.035	0.145	0.078	0.143	0.122	0.3202
250	—	0.355	0.147	—	0.157	0.752
300	0.037	0.558	0.232	0.160	0.176	0.755
400	0.056	—	—	0.233	—	—
500	0.062	—	—	0.300	—	—
600	0.077	—	—	0.417	—	—
700	0.081	—	—	0.569	—	—
800	0.093	—	—	0.664	—	—
900	0.109	—	—	0.749	—	—
1000	0.138	—	—	0.773	—	—

“—”: Not determination.

### Analysis of polysaccharides

#### Analysis of zinc content

The content of zinc in MPS and MZPS were analyzed. The zinc content in MZPS (2.95 ± 0.05 mg/g) was markedly higher than that in MPS (0.62 ± 0.03 mg/g) (P < 0.01).

#### Evaluation of gas chromatogram

**Figure 2:**
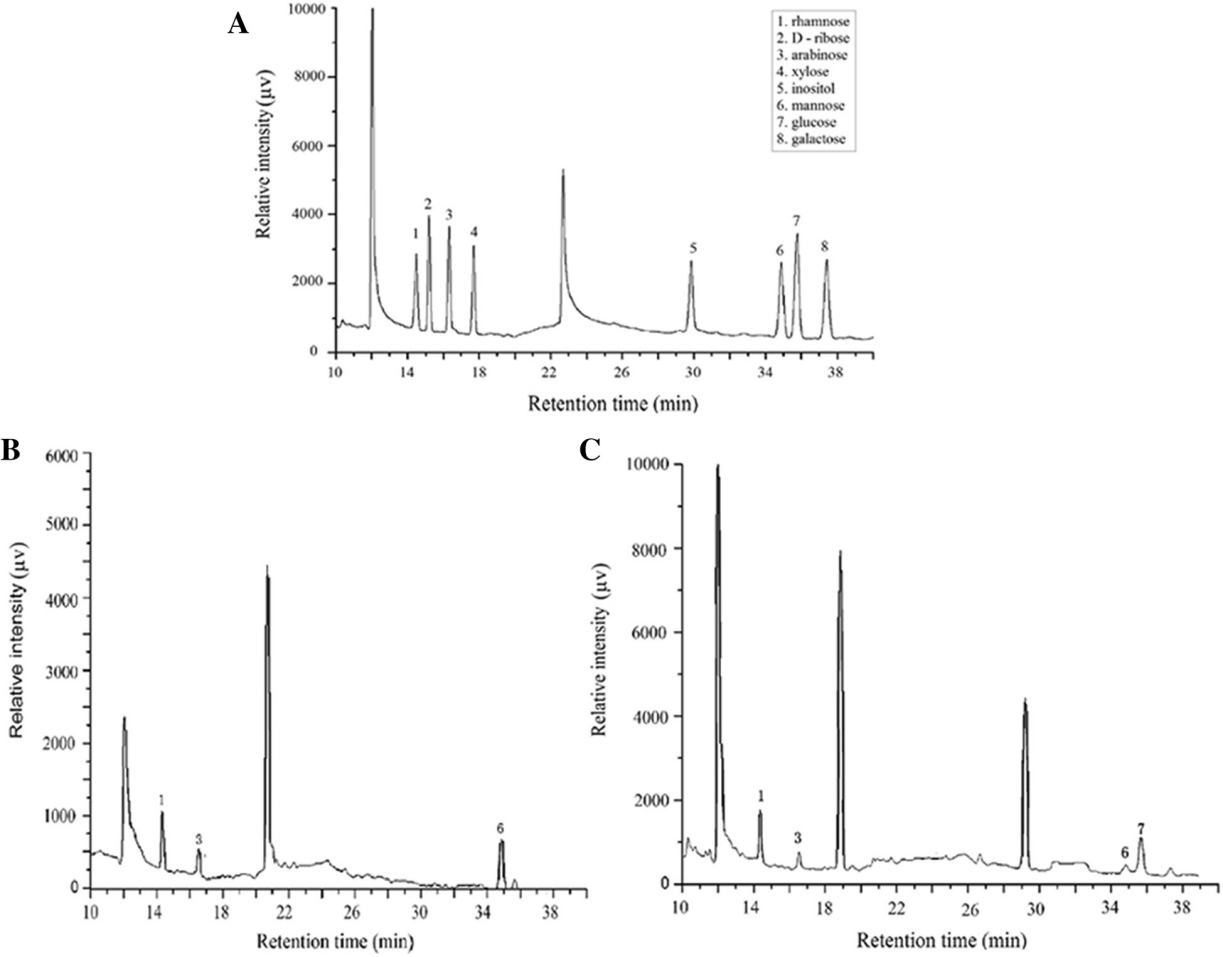
**Gas chromatogram of polysaccharide**. **(A)** chromatogram of standard. **(B)** chromatogram of MPS. **(C)** chromatogram of MZPS.

Gas chromatographic figure of mixed standard monosaccharides, MPS and MZPS contained many peaks as shown in Figure 2. Gas chromatography analysis showed that there were several monosaccharide compositions and proportions in MPS and MZPS. MPS was composed of rhamnose, arabinose and mannose with a ratio of 1.75:1.00:3.02, and MZPS contained rhamnose, arabinosemannose and glucose with a ratio of 7.19:2.26:1.00:8.39, respectively.

#### Analysis of molecular weight

**Figure 3:**
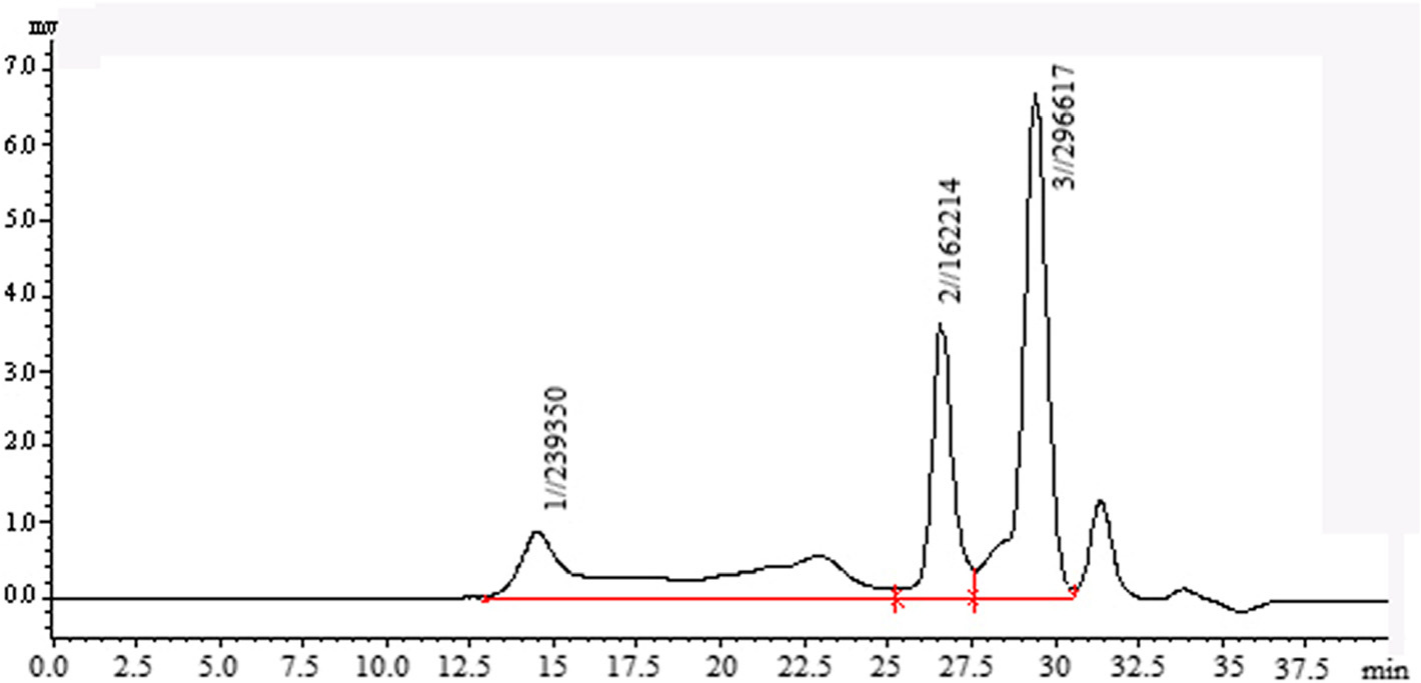
**HPGPC chromatogram of MZPS.**

HPGPC chromatogram (Figure 3) indicated that the Mw (weight-average molecular weight), Mn (number-average molecular weight) and Mz (z-average molecular weight) MZPS were 1.20 × 10^5^, 7.14 × 10^2^ and 7.56 × 10^5^ Da, respectively. The Mw/Mn value of MZPS was 168.19 and the Mz/Mw value was 6.30.

#### Infrared spectral analysis of polysaccharides

**Figure 4:**
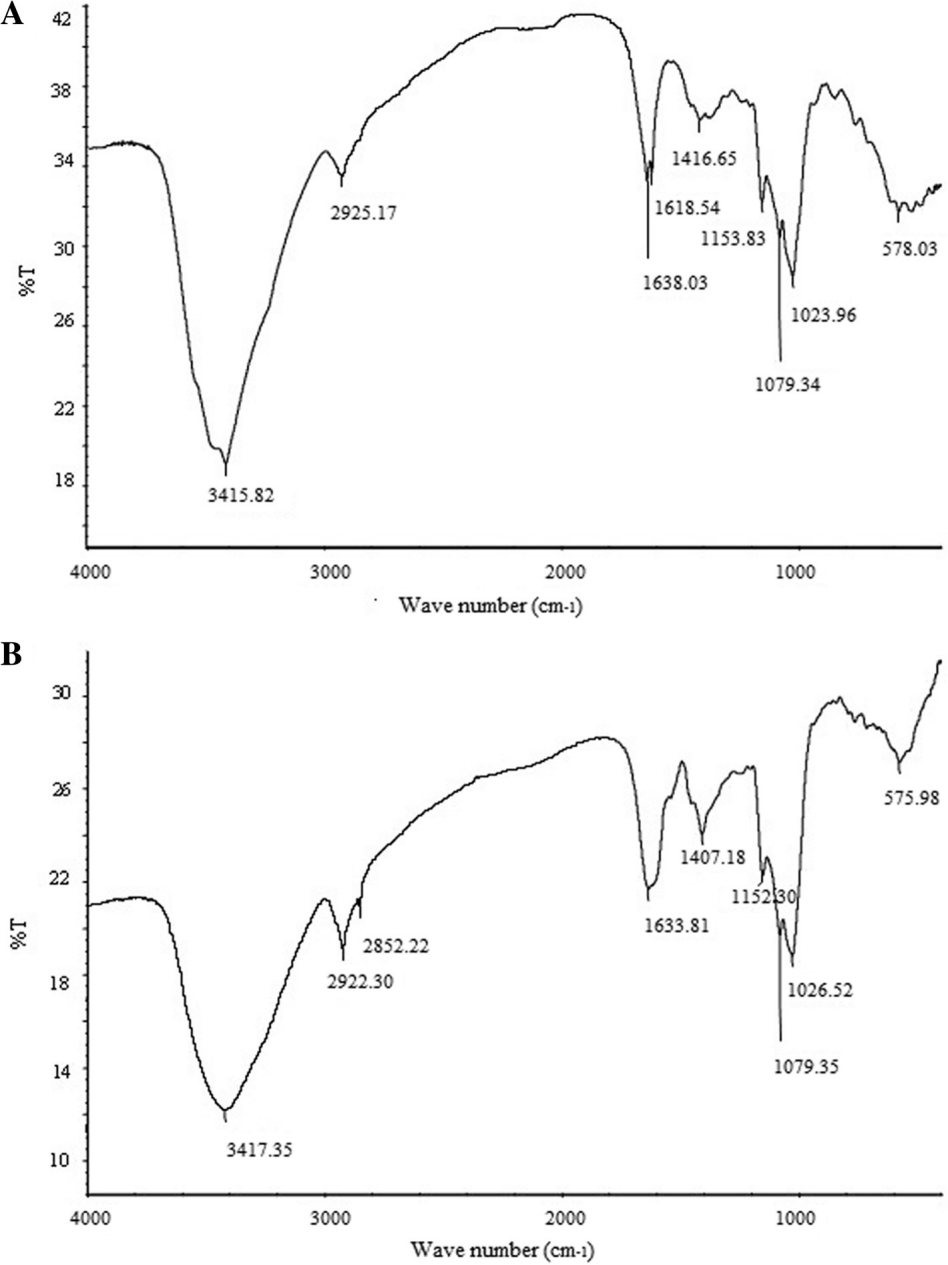
**Infrared spectrum**. **(A)** MPS and **(B)** MZPS.

Infrared spectrum of the MPS and MZPS, as shown in Figure 4, displayed a broadly-stretched intense peak at 3415.82 cm^-1^ and 3417.35 cm^-1^ characteristic of hydroxyl groups and a weak C-H band at around 2925.17 cm^-1^ and 2922.30 cm^-1^. The relatively strong absorption peak at 1638.03 cm^-1^ and 1633.81 cm^-1^ (around 1600-1650 cm^-1^) indicated the existing of C=O, the peaks at 578.03 cm^-1^ and 575.98 cm^-1^ suggestted the presence of -CH_2_ [30]. Characterization of MPS and MZPS by IR analysis showed the typical absorption of pyranose ring at 1153.83 cm^-1^, 1079.34 cm^-1^, 1023.96 cm^-1^ and 1152.30 cm^-1^, 1079.35 cm^-1^, 1026.52 cm^-1^, respectively. In addition, MPS had an absorption peak at 1618.54 cm^-1^ characteristic of -NH_3_^+^ and -NH_2_, showing that MPS was a protein-bound polysaccharide. The absorption peak at 1416.65 cm^-1^ indicated that the characteristic of -COOH in MPS. The absorption peaks at 2852.22 cm^-1^ and 1407.18 cm^-1^ indicated that MZPS was an aliphatic polysaccharide and had a C-H band.

### Anti-aging *in vivo* of MPS and MZPS

In our present work, we built the senile model using D-galactose. Since free-radical-induced lipid peroxidation has been associated with a number of diseases [31], free-radical scavenging capacity detection is our target. The experimental data are shown in Table 4.

**Table 4:** **Effects of MPS and MZPS on the activities of T-AOC, GSH-Px and SOD in heart, liver, kidney [U/mg] and blood [U/mL] and MDA in heart, liver, kidney [nmol/g] and blood [nmol/mL] of aging mice** ^**λ**^

	**CK**	**Model**	**MPS**		**MZPS**	
			**LMPS**	**HMPS**	**LMZPS**	**HMZPS**
T–AOC						
Heart	25.65 ± 0.01	1.23 ± 0.01	5.49 ± 0.50^bd^	19.29 ± 0.51^bd^	8.42 ± 0.01^bd^	25.01 ± 0.01^bd^
Liver	26.04 ± 0.01	5.94 ± 0.01	7.04 ± 0.01^bd^	25.53 ± 0.01^bd^	13.04 ± 0,07^bd^	26.04 ± 0.01^bd^
Kidney	17.83 ± 0.01	5.81 ± 0.01	6.76 ± 0.01^bd^	14.94 ± 6.35	8.37 ± 0.01^bd^	15.23 ± 0.10^bd^
Blood	21.09 ± 0.01	5.48 ± 0.01	6.29 ± 0.01^bd^	18.63 ± 0.01^bd^	8.97 ± 0.01^bd^	20.39 ± 0.01^bd^
GSH–Px						
Heart	0.093 ± 0.02	0.006 ± 0.01	0.011 ± 0.01^bd^	0.075 ± 0.01^bd^	0.022 ± 0.01^bd^	0.089 ± 0.01
Liver	0.274 ± 0.09	0.011 ± 0.11	0.012 ± 0.01^b^	0.106 ± 0.01^bd^	0.039 ± 0.01^bd^	0.208 ± 001^bd^
Kidney	0.167 ± 0.02	0.014 ± 0.03	0.041 ± 0.01^bd^	0.087 ± 0.01^bd^	0.066 ± 0.01^bd^	0.131 ± 0.01^bd^
Blood	0.217 ± 0.07	0.149 ± 0.05	0.154 ± 0.04^bd^	0.191 ± 0.02^bd^	0.174 ± 0.03^bd^	0.208 ± 0.02^bd^
SOD						
Heart	632.20 ± 2.55	342.66 ± 0.57	376.22 ± 8.24^bd^	568.55 ± 0.51^d^	398.22 ± 0.38^bd^	633.11 ± 0.69^d^
Liver	307.25 ± 1.09	133.68 ± 3.05	144.81 ± 0.73^bd^	263.08 ± 1.01^bd^	243.11 ± 2.77^bd^	302.25 ± 1.40^bd^
Kidney	14.74 ± 0.25	1.61 ± 0.12	2.18 ± 0.74^b^	13.19 ± 0.73^ad^	8.14 ± 0.25^bd^	14.24 ± 0.42^d^
Blood	303.08 ± 3.51	64.77 ± 0.39	74.96 ± 0.94^bd^	269.08 ± 1.01^bd^	113.24 ± 1.08^bd^	290.28 ± 6.03^ad^
MDA						
Heart	1.13 ± 0.01	3.63 ± 0.01	3.44 ± 0.01^bd^	1.34 ± 0.01^bd^	2.77 ± 0.01^bd^	1.21 ± 0.01^bd^
Liver	0.36 ± 0.02	3.40 ± 0.03	3.33 ± 0.01^bd^	0.54 ± 0.01^bd^	2.98 ± 01.01^bd^	0.43 ± 0.01^bd^
Kidney	0.03 ± 0.02	0.09 ± 0.01	0.09 ± 0.01^bd^	0.06 ± 7.51^bd^	0.38 ± 0.01^bd^	0.04 ± 0.01^bd^
Blood	1.03 ± 0.01	2.86 ± 0.02	2.72 ± 0.01^bd^	1.25 ± 0.01^bd^	2.53 ± 0.01^bd^	1.03 ± 0.01^bc^

λ: Values are expressed as Mean ± S.D. [Std. Deviation, n = 5]. Paired Samples Statistics [*T–T* test] are used for the significant difference compare to the CK or Model.

Differences are considered significant at *P* < 0.05.

a: Significant difference compare to CK; *P* < 0.05.

b: Significant difference compare to CK; *P* < 0.01.

c: Significant difference compare to Model; *P* < 0.05.

d: Significant difference compare to Model; *P* < 0.01.

The T-AOC activities of the mice in MZPS were significantly lower than those of the mice in MPS. The T-AOC activities of HMZPS reached 25.01 ± 0.01 U/mg, 26.04 ± 0.01 U/mg, 15.23 ± 0.10 U/mg and 20.39 ± 0.01 U/mL in heart, liver, kidney and blood, which were 16.59 U/mg, 13 U/mg, 6.86 U/mg and 11.42 U/mL higher than that of LMZPS, respectively. Moreover, The T-AOC activities of HMZPS in heart, liver, kidney and blood were 5.72 U/mg, 0.51 U/mg, 0.29 U/mg and 1.76 U/mL higher than that of HMPS, respectively. The results indicated that HMZPS had the highest activity among the four groups (LMPS, HMPS, LMZPS and HMZPS).

Significant increases in GSH-Px activities were observed between MZPS and MPS. The GSH-Px activities of HMZPS were increased by 0.07 U/mg, 0.17 U/mg, 0.07 U/mg and 0.03 U/mL than that of LMZPS, and by 0.01 U/mg, 0.10 U/mg, 0.04 U/mg and 0.02 U/mL than that of HMPS, respectively. The results indicated that the MZPS had the higher activities of GSH-Px.

Results showed that the SOD activities rose in a dose-dependent manner. The SOD activities of HMZPS reached 633.11 ± 0.69 U/mg, 302.25 ± 1.40 U/mg, 14.24 ± 0.42 U/mg and 290.28 ± 6.03 U/mL in heart, liver, kidney and blood, which were increased by 234.89 U/mg, 59.14 U/mg, 6.1 U/mg and 177.04 U/mL than that of LMZPS. Compared to HMPS, the SOD activities of HMZPS increased 64.56 U/mg, 39.17 U/mg, 1.05 U/mg and 21.20 U/mL, respectively.

The MDA contents of HMZPS reached 1.21 ± 0.01 nmol/g, 0.43 ± 0.01 nmol/g, 0.04 ± 0.10 nmol/g and 1.03 ± 0.01 nmol/mL in heart, liver, kidney and blood, which were 1.56 nmol/g, 2.55 nmol/g, 0.34 nmol/g and 1.50 nmol/mL (all P < 0.01) lower than that of LMZPS, respectively, and 0.13 nmol/g 0.11 nmol/g, 0.02 nmol/g and 0.22 nmol/mL lower than that of HMPS, respectively. The results indicated that MZPS had ability of lowering lipid contents in mice tissue.

## Discussions

In the present work, the preliminary chemical characterization of MZPS was determined. The results indicated that the major component of MZPS was glucose, and the Mw of MZPS was 1.20 × 10^5^ Da. Li et al. [32] also found that glucose was the most abundant monosaccharide in the Lentinan. However, it has been reported that the main polysaccharide extracted from *L. edodes* fruiting bodies was composed of arabinose, galactose, glucose, xylose, and mannose with a molar ratio of 0.72:3.01:33.77:1.34:3, and the Mw was 1.06 × 10^6^ Da [33]. Therefore, the structural characterization of MZPS was different from that of fruit body polysaccharide of *L. edodes*.

We measured various antioxidant activities *in vitro* of MPS and MZPS and their four fractions. Free radicals are known to be the major cause of various chronic and degenerative diseases, including aging, coronary heart disease, inflammation, stroke, diabetes mellitus and cancer [34]. The antioxidant compounds play an important role in preventing and curing chronic inflammation, atherosclerosis, cancer and cardiovascular disorders [35]. Reductant can quench free radicals by transmitting electrons to them. The electron-donating potential of a given compound, termed as reducing capacity, may serve as a significant indicator of its potential antioxidant activity [36]. Hydroxyl radicals are main reactive oxygen free radicals in living organisms, can severely damage adjacent biomolecules such as proteins, DNA, fatty acids and nucleic acids [37], they are the significant causes for causing the general pathological processes of aging and tissue damage, and could influence the evolution of many degenerative diseases [38]. DPPH is one of the few stable and commercially available organic nitrogen radicals which can accept an electron or hydrogen atom to become a stable diamagnetic molecule [39]. When DPPH encounters a proton-donating substance such as an antioxidant, the radical would be scavenged and the absorbance is reduced [40]. It has been widely used as a substrate to test the radical-scavenging ability of various samples. The antioxidant activity of MZPS-2 was higher than that of other three fractions, but lower than that of MZPS. The possible mechanism may be that the interaction of the two fractions promoted the improvement of the antioxidant activity of MZPS. Detailed analysis would be possessed in further studies.

We also measured various anti-aging effects *in vivo* of MPS and MZPS. In this work, markedly decreased T-AOC, GSH-Px and SOD activity, increased MDA levels were found in the Model group. The MPS and MZPS could significantly counteract the increased oxidative stress by promoting the activities of enzymatic and non-enzymatic antioxidants and reducing the level of lipid peroxidative product after fed to the mice. These experimental data indicated that the MPS and MZPS have antioxidant activity and a potential for decreasing risk of aging.

## Conclusion

MZPS effectively showed anti-aging activities *in vivo* and antioxidant activities *in vitro*, and the molecular constituents, chemical bonds and functional groups of MZPS were different from that of MPS, suggesting that the MZPS of *L. edodes* SD-08 could be used as a potentially natural antioxidant. However, the relationship of structure and activity of MZPS and its possible mechanisms were the areas for future studies.

## Abbreviations

MPS: Mycelia polysaccharides
MZPS: Mycelia zinc polysaccharides
PDA: Potato dextrose agar
DPPH: 1, 1-dipheny-l,2-picrylhydrazyl
MG: Model group
BG: Blank group
LMPS: Low MPS
HMPS: High MPS
LMZPS: Low MZPS
HMZPS: High MZPS
T-AOC: Total antioxidant capacity
SOD: Superoxide dismutase
GSH-Px: Glutathione peroxidase
MDA: Malonaldehyde
NADPH: Nicotinamide adenine dinucleotide phosphate.

## References

[1] Carbonero ER, Gracher AHP, Komura DL, Marcon R, Freitas CS, Baggio CH (2008). *Lentinus edodes* heterogalactan: antinociceptive and anti-inflammatory effects. Food Chem.

[2] Surenjav U, Zhang L, Xu XJ, Zhang XF, Zeng FB (2006). Effects of molecular structure on antitumor activities of [1 → 3]-β-D-glucans from different *Lentinus edodes*. Carbohydr Polym.

[3] Hatvani N (2001). Antibacterial effect of the culture fluid of *Lentinus edodes* mycelium grown in submerged liquid culture. Int J Antimicrob Agents.

[4] Jong SC, Birmingham JM (1993). Medicinal and therapeutic value of the shiitake mushroom. Adv Appl Microbiol.

[5] Ren R, Ma HL, Liu B, Zhao WR (2008). Study on the degradation of Lentinan by ultrasonication and its antioxidant activity *in vitro*. J Anhui Agr Sci.

[6] Ikekawa T, Uehara N, Maeda Y, Nakanishi M, Fukuoka F (1969). Anti–tumor activity of aqueous extracts of edible mushrooms. Cancer Res.

[7] Chihara G (1992). Immunopharmacology of lentinan, a polysaccharide isolated from *Lentinus edodes*: its application as a host defense potentiator. Int J Ori Med.

[8] Fabris N (1992). Biomarkers of aging in the neuroendocrine-immune domain. Time for a new theory of aging. Ann NY Acad Sci.

[9] Fabris N, Mocchegiani E, Muzzioli M, Provinciali M (1991). The role of zinc in neuroendocrine-immune interactions during aging. Ann NY Acad Sci.

[10] Vallee BL (1977). Zinc biochemistry in normal and neoplastic growth processes. Experientia.

[11] Hambidge KM, Itambidge C, Jacobs M, Baum JD (1972). Low levels of zinc in hair, anorexia, poor growth, and hypogeusia in children. Pediatr Res.

[12] Maret W, Sandstead HH (2006). Zinc requirements and the risks and benefits of zinc supplementation. J Trace Elem Med Biol.

[13] Chaplin MF, Kennedy JF. Carbohydrate analysis, a practical approach. Oxford University Press. 1994, 1:1–41

[14] Tan RX (2002). Analysis of plant composition.

[15] Oyaizu M (1980). Studies on products of browning reaction: antioxidative activities of products of browning reaction prepared from glucosamine. Jpn J Nutr.

[16] Winterbourn CC, Sutton HC (1984). Hydroxyl radical production from hydrogen peroxide and enzymatically generated paraquat radicals: catalytic requirements and oxygen dependence. Arch Biochem Biophys.

[17] Liu XN, Zhou B, Lin RS, Jia L, Deng P, Fan KM (2010). Extraction and antioxidant activities of intracellular polysaccharide from *Pleurotus sp.* mycelium. Int J Biol Macromol.

[18] Sheng JC, Yu F, Xin ZH, Zhao LY, Zhu XJ, Hu QH (2007). Preparation, identification and their antitumor activities in vitro of polysaccharides from *Chlorella pyrenoidosa*. Food Chem.

[19] Kumar CG, Joo HS, Choi JW, Koo YM, Chang CS (2004). Purification and characterization of an extracellular polysaccharide from haloalkalophilic *Bacillus* sp. I-450. Enzyme Microb Technol.

[20] Flohé L, Günzler WA (1984). Assays of glutathione peroxidase. Methods Enzymol.

[21] Zhao SJ (2002). Experiment instruction of plant physiology.

[22] Cheng HY, Lin TC, Yu KH, Yang CM, Lin CC (2003). Antioxidant and free radical scavenging activities of *Terminalia chebula*. Biol Pharm Bull.

[23] Kohen R, Nyska A (2002). Oxidation of biological systems: oxidative stress phenomena, antioxidants, redox reactions, and methods for their quantification. Toxicol Pathol.

[24] Xie LY, Zhang Y, Peng WH, Gan BC (2011). Immune function and antioxidant activity of intracellular polysaccharides from *Phellinus baumii*. Food Sci.

[25] Xie LY, Zhang Y, Guo Y, Wu X, Peng WH, Gan BC (2011). Isolation, purification and physico-chemical characteristics analysis of mycelial polysaccharides from *Phellinus baumii*. Food Sci.

[26] Reis FS, Martins A, Barros L, Ferreira IC (2012). Antioxidant properties and phenolic profile of the most widely appreciated cultivated mushrooms: a comparative study between *in vivo* and *in vitro* samples. Food Chem Toxicol.

[27] Ajibola CF, Fashakin JB, Fagbemi TN, Aluko RE (2011). Effect of peptide size on antioxidant properties of African yam bean seed [*Sphenostylis stenocarpa]* protein hydrolysate fractions. Int J Mol Sci.

[28] Aruoma OI (1998). Free radicals. oxidative stress, and antioxidants in human health and disease. J Am Oil Chem Soc.

[29] Li YQ, Wu J, Hua LM, Wu YW, Yang QZ, Zhang YK (2003). Antioxidative activity of polysaccharide from *Pleurotus nebrodensis* mycelium. J Lanzhou Univ.

[30] Hu YL, Chen HW, Zhang C (2011). Scavenging function of intracellular polysaccharide of enriched selenium *Cordyceps militaris* on radical. Agr Eng.

[31] Wang D, Wang W, Mou HJ, Wang J, Teng LR, Zhang YB (2009). Optimization of ultrasound-assisted extraction of *Tricholoma matsutake* mycelium polysaccharide using response surface methology and its antioxidative effect. Chem Ind Forest Prod.

[32] Li J, Liu N, Chen P, Yan EZ, An GJ (2005). Determination method of composition and content of monosaccharide in the mushroom polysaccharide. Chem Adhes.

[33] Yang J, Wu MC, Zhang SH (2001). Isolation, purification and characterization of polysaccharides from the fruitbody of *Lentinus edodes* (Berk.). Sing J Plant Res Envir.

[34] Jayakumar T, Thomas PA, Geraldine P (2011). *In-vitro* and *in-vivo* antioxidant effects of the oyster mushroom *Pleurotus ostreatus*. Food Res Int.

[35] Shimada K, Fujikawa K, Yahara K, Nakamura T (1992). Antioxidative properties of xanthan on the antioxidation of soybean oil in cyclodextrin emulsion. J Agr Food Chem.

[36] Soares JR, Dinis TCP, Cunha AP, Almeida LM (1997). Antioxidant activities of some extracts of *Thymus zygis*. Free Radical Res.

[37] Zhu YL, Chen HW, Zhang C (2011). Scavenging function of intracellular polysaccharide of enriched selenium *Cordyceps militaris* on radical. Agr Eng.

[38] Li XL, Zhao J, Li D, Gao CC (2009). Comparative study on antioxidant activities of mycelium from submerged fermentation of *Cordyceps sinensis* [Berk.] and fruit body from cultured *Cordyceps militaris* [L.] Link. Food Sci Technol.

[39] Kacurakova M, Capek P, Sasinkova V, Wellner N, Ebringerova A (2000). FT-IR study of plant cell wall model compounds: pectic polysaccharides and hemicelluloses. Carbohydr Polym.

[40] Xiao N, Wang XC, Diao YF, Liu R, Tian KL (2004). Effect of initial fluid resuscitation on subsequent treatment in uncontrolled hemorrhagic shock in rats. Shock.

